# The frequent *BRCA1 *mutation 1135insA has multiple origins: a haplotype study in different populations

**DOI:** 10.1186/1471-2350-7-15

**Published:** 2006-03-01

**Authors:** Teresa M Rudkin, Nancy Hamel, Maria Galvez, Frans Hogervorst, Johan JP Gille, Pål Møller, Jaran Apold, William D Foulkes

**Affiliations:** 1 Department of Human Genetics, McGill University, Montreal, Canada; 2 Department of Medicine and Research Institute, McGill University Health Centre, Montreal, Canada; 3 Family Cancer Clinic, Department of Pathology, The Netherlands Cancer Institute, Amsterdam, The Netherlands; 4 Department of Clinical Genetics, VU University Medical Center, Amsterdam, The Netherlands; 5 Department of Cancer Genetics, The Norwegian Radium Hospital, N-0310 Oslo, Norway; 6 Center of Medical Genetics and Molecular Medicine, Haukeland University Hospital, Bergen, Norway; 7 Cancer Prevention Centre, Sir Mortimer B Davis-Jewish General Hospital, McGill University, Montreal, Canada

## Abstract

**Background:**

Analysis of the chromosomal background upon which a mutation occurs can be used to reconstruct the origins of specific disease-causing mutations. The relatively common *BRCA1 *mutation, 1135insA, has been previously identified as a Norwegian founder mutation. We performed haplotype analysis of individuals from breast and ovarian cancer families from four different ethnic backgrounds who had been identified as carriers of the *BRCA1*: 1135insA mutation.

**Methods:**

Four microsatellite markers (D17S855, D17S1322, D17S1323 and D17S1325) located within or near the *BRCA1 *gene were genotyped in mutation carriers from 6 families of French Canadian, Italian and Dutch descent. Haplotypes were inferred from the genotype data and compared between these families and with the previously reported Norwegian founder haplotype.

**Results:**

The 1135insA mutation was found to occur on three distinct haplotype backgrounds. The families from Norway shared a distinct haplotype while the families of French Canadian, Italian, and Dutch descent were found to occur on one of two additional, distinct backgrounds.

**Conclusion:**

Our results indicate that while the Norwegian haplotype including 1135insA represents an ancient Norwegian mutation, the same mutation has occurred independently in the other populations examined. In centres where targeted mutation testing is performed, exclusively or prior to gene sequencing, our findings suggest that this recurring mutation should be included in targeted mutation panels, irrespective of the ethnic origin of the persons tested.

## Background

Analysis of the chromosomal background upon which a mutation occurs can be used to reconstruct the origins of specific disease-causing mutations. Mutations that are seen repeatedly on a common haplotype are likely to have descended from a common ancestor, and are referred to as "founder mutations". Several founder mutations of the breast and ovarian cancer susceptibility gene *BRCA1 *have been identified in individuals of many different ancestries, including families of Ashkenazi Jewish (187delAG, 5385insC) [1], French Canadian (C4446T, 2953del3+C) [2,3], Dutch (2804delAA and Alu-mediated deletions encompassing exons 13 and 22) [4,5], and Polish (5385insC, C61G, 4153delA) [6] origins. The *BRCA1 *mutation 1135insA (HGVS nomenclature: c.1135_1136insA) is a frameshift mutation occurring in exon 11. It has previously been identified as one of four founder mutations originating from the Eastern population of Norway [7,8]. In one series, approximately 1% of Norwegian women under the age of 70 years with ovarian cancer carried this mutation [9] and it accounts for ~20% of all *BRCA1/2 *mutation carriers demonstrated by DNA testing today in Norway (Møller, unpublished data). This mutation appears on an ancient haplotype, and the age of the 1135insA mutation has not yet been determined. All tested Norwegian individuals with the 1135insA mutation carry the same flanking markers (Møller, unpublished data).

In contrast to other Norwegian founder mutations, the 1135insA mutation has also been reported in other ethnic groups. The mutation has been catalogued 44 times to date in the Breast Cancer Information Core (BIC) database [10] and has been reported to occur in populations throughout Europe including Spain, Norway, the Netherlands, Austria, Italy, as well as in Latin America and North America. These observations raise the question whether *BRCA1*: 1135insA is an ancient mutation that has arisen once, or whether it has occurred several times in human history. The issue is relevant when ethnic-specific mutation panels are used as a pre-screen prior to complete exon-by-exon evaluation of *BRCA1/2*. To address this question, we performed haplotype analysis of the *BRCA1 *region in unrelated carrier families of Norwegian, Italian, French Canadian, and Dutch descent.

## Methods

Families containing individuals carrying the *BRCA1*:1135insA mutation were recruited into the study via high-risk cancer genetics clinics at McGill University, Montreal, Quebec, Canada, VU University Medical Center and the National Cancer Institute, Amsterdam, The Netherlands, and The Department of Cancer Genetics, Olso, Norway. Appropriate institutional informed consent guidelines were followed for all recruited patients. All mutation identification was performed by direct sequencing in the laboratories of the primary investigators at McGill University, Montreal; the National Cancer Institute, Amsterdam and Haukeland University Hospital, Bergen. Polymorphic microsatellite repeat markers located within (D17S855, D17S1322, D17S1323) and adjacent to (D17S1325) the *BRCA1 *locus were used for haplotype analysis. We amplified 100ng of genomic DNA from each subject by PCR for each marker using radioactively labelled nucleotides (primer sequences, marker positions and PCR conditions are given in Table 2). PCR products were separated on 5% acrylamide/urea denaturing gels at 70W for 1h45 and alleles were visualized by autoradiography.

**Table 1:** Haplotype analysis of unrelated, *BRCA1 *1135insA carriers

**Marker**	Norway	B856	B050	NKI	Mon270	MonDS	Mon1467
**D17S1325**	218	216	216	216	216	198	198
**D17S1323**	162	158	158	158	158	150	150
**D17S1322**	131	125	125	125	125	122	122
**D17S855**	150	146	146	146	146	150	150

Norway: Six Norwegian families [8]

B856, B050, NK1: Dutch families (Amsterdam)

Mon270, MonDS: Italian families (Montreal)

Mon1467: French Canadian (Montreal)

**Table 2:** Primers used to generate the haplotype

**Marker**	**Chromosome 17 position**	**Forward Primer**	**Reverse Primer**	**T_m _(°C)**	**Other**
D17S855	38,458,470	GGATGGCCTTTTAGAAAGTGG	ACACAGACTTGTCCTACTGCC	57	5 % DMSO†
D17S1322	38,465,161	CTAGCCTGGGCAACAAACGA	GCAGGAAGCAGGAATGGAAC	60	--
D17S1323	38,491,793	TAGGAGATGGATTATTGGTG	AAGCAACTTTGCAATGAGTG	60	--
D17S1325	38,891,165	AAAGGTGGCAATTCACAGTTG	GTGATAAAACTCAGTGGTACTC	65-55*	1× Q solution

All reactions were amplified for 35 cycles [30s@95°C, 30s@T_m_, 30s@72°C] and contained 200 uM dCTP/dGTP/dTTP, 24 uM dATP, 10 uCi α^35^S-dATP, 1× PCR buffer and 0.5 U of HotStar Taq DNA polymerase (QIAGEN). †Dimethyl Sulfoxide. *T_m _starting at 65°C and decreasing by 1°C per cycle for 10 cycles, followed by 25 cycles at 55°C; Q solution from QIAGEN.

## Results

### Clinical description

Haplotype analysis was performed on selected cases from three families recruited from the Hereditary Cancer Clinic of McGill University in Montreal (2 Italian, 1 French Canadian), and three Dutch families from the Netherlands Cancer Institute (NKI) in Amsterdam. Haplotype analysis was previously done in Norway on six families [8]. The index patients were referred because of a personal and/or family history of breast or ovarian cancer and were subsequently found to be carriers of the *BRCA1*: 1135insA mutation. Although not discussed above, the family histories of these cases are consistent with this mutation being highly penetrant for both breast and ovarian cancer. The family histories of the Norwegian families have been described previously [8].

### Haplotype analysis

The results of the haplotype analyses are shown in Table 1. The 1135insA mutation was found to occur on three distinct haplotype backgrounds. The families from Norway had a unique haplotype (218-162-131-150). The three Dutch families shared a common haplotype (216-158-125-146), which was also found in one Italian family from Montreal. The remaining two families from Montreal – one family of French Canadian descent and the other of Italian descent – shared a third distinct haplotype (198-150-122-150).

## Discussion

The unique haplotype common to all 6 Norwegian families (218-162-131-150) indicates 1135insA is an ancient founder mutation in the Norwegian population. The Netherlands contained a major port during the Middle Ages with outposts to ports elsewhere in Europe. By the seventeenth century, Amsterdam was the biggest trading port of northern commodities and grain to the Mediterranean [12]. One can hypothesize that these trade routes are one means by which a Norwegian founder mutation, on its original haplotype, may have been passed to various Mediterranean countries and hence to North America. Identification of two other, clearly distinct haplotypes in the families examined here suggests, however, that the mutation likely occurred independently in other populations.

In the BIC database [10], the 1135insA mutation is the 12^th ^most common frameshift mutation occurring in *BRCA1*. Since our results suggest it does not have a single origin, it may be a relative "hot spot" within the *BRCA1 *gene. Recombination events or marker mutations could theoretically account for haplotype divergence from a single, original haplotype but are not likely explanations here. Marker D17S855 in intron 20 is only 6.7 kb from D17S1322 in intron 19, and D17S1322 is 26 kb from D17S1323 in intron 12. Markers in such close proximity are unlikely to undergo extensive recombination events. While recombination could conceivably have occurred between D17S1325, located 400 kb upstream from D171323, and the intragenic markers, the haplotypes observed in our families are not consistent with this scenario. In particular, recombination suppression seems to be present in the genomic region that includes *BRCA1*, making recombination in this region an even more unlikely explanation [13]. On the other hand, the DNA sequence surrounding the mutation includes a run of seven consecutive adenines. *BRCA1*:1135insA is an insertion of one A anywhere in the poly-A area in which it occurs – the position 1135 is but a convention for nomenclature. This poly-A region may thus create a hot-spot within *BRCA1 *for replication errors. Alternatively, the same poly-A stretch may have induced repetitive gene conversions or cross-overs.

The *BRCA2 *mutation 8765delAG is another example of an "ancient" mutation that has been shown to have recurred in different populations. First described as a founder mutation in Sardinia [14], it has been found on different haplotype backgrounds in different populations, including French Canadians and Yemenite Jews [15]. The mutation occurs within a stretch of DNA where 4 consecutive AGs can be found. It is likely that the genomic context in which the *BRCA1*: 1135insA and *BRCA2*: 8765delAG mutations occur favours errors of small insertions and deletions.

## Conclusion

We set out to determine whether *BRCA1*: 1135insA is an ancient mutation that has arisen once, or whether it has recurred several times in human history. Our finding that 1135insA exists on several, clearly distinct haplotypes and the fact that it is found in a homopolymer tract suggests it may have appeared independently several times in the human genome. A clinically relevant implication of our findings is that this recurrent mutation should probably be included in targeted *BRCA1 *mutation screening panels in any population, irrespective of ethnic origin. When the ethnic origin is clearly defined, founder mutation panels can, and are used in several centres. A "pre-screen" based on the most frequent *BRCA1/2 *mutations seen in the BIC could also be considered as a first step, prior to complete gene sequence analysis.

## Competing interests

The author(s) declare that they have no competing interests.

## Authors' contributions

TMR wrote the drafts and produced the final manuscript. NH did most of the genotyping and haplotype reconstruction and assisted in writing the manuscript. MG carried out some of the genotyping. FH did some genotyping and contributed to the writing of the manuscript. JJPG provided cases. PM provided cases and assisted in the writing of the manuscript. JA provided cases and provided comment on the manuscript. WDF conceived of and managed the project, provided cases, and assisted in drafting the manuscript. All authors read and approved the final manuscript.

## Pre-publication history

The pre-publication history for this paper can be accessed here:


